# Uniaxial polarization analysis of bulk ferromagnets: theory and first experimental results^1^

**DOI:** 10.1107/S1600576722003508

**Published:** 2022-05-28

**Authors:** Artem Malyeyev, Ivan Titov, Charles Dewhurst, Kiyonori Suzuki, Dirk Honecker, Andreas Michels

**Affiliations:** a Department of Physics and Materials Science, University of Luxembourg, 162A Avenue de la Faïencerie, 1511, Luxembourg; b Institut Laue–Langevin, 6 Rue Jules Horowitz, BP 156, F-38042 Grenoble Cedex 9, France; c Department of Materials Science and Engineering, Monash University, Clayton, Victoria 3800, Australia; d ISIS Neutron and Muon Source, Rutherford Appleton Laboratory, Didcot, United Kingdom

**Keywords:** polarized neutron scattering, uniaxial polarization analysis, small-angle neutron scattering, micromagnetics, magnetic nanocomposites

## Abstract

On the basis of Brown’s static equations of micromagnetics, the uniaxial polarization of the scattered neutron beam of a bulk magnetic material is computed. The approach considers a Hamiltonian that takes into account the isotropic exchange interaction, the antisymmetric Dzyaloshinskii–Moriya interaction, magnetic anisotropy, the dipole–dipole interaction and the effect of an applied magnetic field. In the high-field limit, the solutions for the magnetization Fourier components are used to obtain closed-form results for the spin-polarized small-angle neutron scattering (SANS) cross sections and the ensuing polarization. The theoretical expressions are compared with experimental data on a soft magnetic nanocrystalline alloy. The micromagnetic SANS theory provides a general framework for polarized real-space neutron methods, and it may open up a new avenue for magnetic neutron data analysis on magnetic microstructures.

## Abstract 2

Based on the continuum theory of micromagnetics, theoretical expressions for the polarization of the scattered neutron beam in uniaxial small-angle neutron scattering have been derived and their predictions tested by analyzing experimental data on a soft magnetic nanocrystalline alloy. The here-presented theoretical framework forms the basis for polarized real-space methods such as spin-echo small-angle neutron scattering, spin-echo modulated small-angle neutron scattering and polarized neutron dark-field contrast imaging.

## 1. Introduction

Polarized neutron scattering is one of the most powerful techniques for investigating the structure and dynamics of condensed matter, in particular magnetic materials and superconductors (Chatterji, 2006 ▸). Based on the seminal papers by Bloch (1936 ▸, 1937 ▸), Schwinger (1937 ▸) and Halpern & Johnson (1939 ▸), the theory of polarized neutron scattering was worked out in the early 1960s by Maleev *et al.* (1963 ▸) and Blume (1963 ▸). Several classic experimental studies (Shull *et al.*, 1951 ▸; Moon *et al.*, 1969 ▸; Rekveldt, 1971 ▸; Drabkin *et al.*, 1972 ▸; Okorokov *et al.*, 1978 ▸; Pynn *et al.*, 1983 ▸; Mezei, 1986 ▸; Schärpf & Capellmann, 1993 ▸) have demonstrated the basic principles and paved the way for today’s three-dimensional cryogenic polarization analysis device (CRYOPAD) (Tasset, 1989 ▸; Tasset *et al.*, 1999 ▸; Brown *et al.*, 1993 ▸; Okorokov & Runov, 2001 ▸). With this technique it becomes possible to measure 16 correlation functions [see the paper by Mezei (1986 ▸) for a detailed discussion], which provide important information on the nuclear and magnetic structure of materials [see Williams (1988 ▸) and Lovesey (1984 ▸) for textbook expositions of polarized neutron scattering].

However, for the scattering of cold (long-wavelength) neutrons along the forward direction – as implemented on a small-angle neutron scattering (SANS) instrument – it has only in recent years become possible to perform neutron polarization analysis (retaining the full two-dimensional scattering information) ‘routinely’: more specifically, uniaxial (also called longitudinal or one-dimensional) polarization analysis, where the polarization of the scattered neutrons is analyzed along the direction of the initial polarization (Moon *et al.*, 1969 ▸). Clearly, this progress is due to the development of efficient ^3^He spin filters (*e.g.* Batz *et al.*, 2005 ▸; Petoukhov *et al.*, 2006 ▸; Okudaira *et al.*, 2020 ▸), which, in contrast to *e.g.* single-crystal analyzers, can be used over a rather broad wavelength range and cover a large detector acceptance angle. Note also that Niketic *et al.* (2015 ▸) and Quan *et al.* (2019*a* ▸,*b* ▸) report on the development of a novel neutron spin filter, based on the strong spin dependence of the neutron scattering on protons. For the combination of uniaxial polarization analysis with SANS, the term POLARIS has been coined (Wiedenmann, 2005 ▸). In contrast to CRYOPAD, which generally demands the sample to be in a zero magnetic field environment, POLARIS allows for the application of large magnetic fields.

The POLARIS method has been successfully employed for studying the superparamagnetic response of concentrated ferrofluids (Wiedenmann, 2005 ▸), proton domains in deuterated solutions (van den Brandt *et al.*, 2006 ▸; Aswal *et al.*, 2008 ▸; Noda *et al.*, 2016 ▸), the multiferroic properties of HoMn_3_ single crystals (Ueland *et al.*, 2010 ▸), the role of nanoscale heterogeneities for the magnetostriction of Fe–Ga alloys (Mudivarthi *et al.*, 2010 ▸; Laver *et al.*, 2010 ▸), local weak ferromagnetism in BiFeO_3_ (Ramazanoglu *et al.*, 2011 ▸), nanometre-sized magnetic domains and coherent magnetization reversal in an exchange-bias system (Dufour *et al.*, 2011 ▸), precipitates in Heusler-based alloys (Benacchio *et al.*, 2019 ▸), the magnetic microstructure of nanoscaled bulk magnets (Honecker *et al.*, 2010 ▸; Michels *et al.*, 2012 ▸), the internal spin structure of nanoparticles (Krycka *et al.*, 2010 ▸, 2014 ▸; Hasz *et al.*, 2014 ▸; Grutter *et al.*, 2017 ▸; Orue *et al.*, 2018 ▸; Bender, Fork *et al.*, 2018 ▸; Bender, Wetterskog *et al.*, 2018 ▸; Oberdick *et al.*, 2018 ▸; Ijiri *et al.*, 2019 ▸; Bender *et al.*, 2019 ▸; Honecker *et al.*, 2020 ▸), and Invar alloys (Stewart *et al.*, 2019 ▸). Polarization analysis further makes it possible to reveal the direction of the magnetic anisotropy in single-crystalline spin systems, *e.g.* an easy plane versus an easy axis anisotropy or the confinement of the propagation vector along certain crystallographic directions in chiral and other exotic magnets (Takagi *et al.*, 2018 ▸; White *et al.*, 2018 ▸). In all of the above-mentioned studies, the spin-resolved SANS cross sections were obtained and analyzed, but the polarization of the scattered neutrons was not further investigated.

Historically, this is the domain of the neutron depolarization technique (see *e.g.* Halpern & Holstein, 1941 ▸; Hughes *et al.*, 1948 ▸, 1949 ▸; Burgy *et al.*, 1950 ▸; Maleev & Ruban, 1970 ▸; Rekveldt, 1971 ▸, 1973 ▸; Drabkin *et al.*, 1972 ▸; Maleev & Ruban, 1972 ▸; Rosman & Rekveldt, 1991 ▸, and references therein), where one measures the change in the polarization of a polarized neutron beam after transmission through a partially magnetized magnetic material. Analysis of the 3 × 3 depolarization matrix yields information on *e.g.* the average domain size and the domain magnetization. This type of polarization analysis on a SANS instrument has been termed ‘vector analysis of polarization’ by Okorokov & Runov (2001 ▸). Alternatively, it has been demonstrated that the neutron spin-echo technique can resolve magnetic small-angle scattering (Grigoriev *et al.*, 2006 ▸; Rekveldt *et al.*, 2006 ▸). Spin-echo small-angle neutron scattering (SESANS) provides information on correlations on a length scale from about 10 nm to 10 µm. In SESANS, the neutron spin precesses in a constant magnetic field and the neutron runtime difference due to sample scattering results in the dephasing of the neutron spins and in a loss of the measured beam polarization. Magnetic scattering can result in neutron spin-flip events that act as an additional optical element reversing the sense of the Larmor precession. The change of the neutron spin due to magnetic scattering can be exploited to study magnetic systems.

More recently, the method of polarized neutron dark-field contrast imaging (DFI) has been introduced for spatially resolved small-angle scattering studies of magnetic microstructures (Valsecchi *et al.*, 2021 ▸). First experimental results on a sintered Nd–Fe–B magnet demonstrated not only that dark-field contrast from half-polarized SANS measurements can be observed but also that it becomes possible to separate and retrieve dark-field contrast for all spin-flip and non-spin-flip channels separately. The polarized DFI method has great potential for analyzing real-space magnetic correlations on a macroscopic length scale, well beyond what can be probed with a conventional SANS instrument. Similarly, first measurements of micrometre-sized magnetic correlations have been performed with an alternative neutron precession technique called spin-echo modulated small-angle neutron scattering (SEMSANS) (Li *et al.*, 2021 ▸). With this setup, the spin manipulations are performed before the sample so that the measurement is not sensitive to large stray fields (related *e.g.* to the sample environment), and it even allows studies under beam depolarizing conditions. The polarization analyzer discriminates the polarization parallel to the analyzing direction, such that the scattering cross sections for the opposite neutron spin state are probed at the sample. The two-dimensional neutron polarization modulation observed on the detector is then integrated to yield the one-dimensional correlation function of the system.

For the above techniques, the analysis of magnetic materials is based on performing a neutron-spin analysis deliberately after the sample. The magnetic spin-flip scattering signal of the sample is utilized as a spin flipper to obtain exclusive sensitivity of the signal on local magnetization components. The projected correlation function is modified with the polarization of the scattered neutrons to reflect the additional phase in precession angles due to the spin-flip event.

In this work, we present a micromagnetic SANS theory for the uniaxial polarization of the scattered neutrons of bulk magnetic materials. The approach has recently been employed to analyze SANS cross sections directly in Fourier space (Michels, 2021 ▸) and is here extended to include the final polarization, which can be measured with a much higher precision than the individual cross sections (Brown, 2006 ▸). The continuum theory of micromagnetics allows one to characterize the large-scale magnetization distribution of polycrystalline magnets, which is determined *e.g.* by magnetic anisotropy and saturation-magnetization fluctuations, antisymmetric exchange, and dipolar stray fields. Since the validity of micromagnetic theory extends to the micrometre regime, the theoretical framework developed here may also serve as a basis for the above-mentioned polarized neutron techniques (SESANS, DFI, SEMSANS), which provide real-space information on large-scale magnetic correlations. Rekveldt *et al.* (2006 ▸) summarize the relevant expressions which relate the magnetization distribution of the material (obtained from micromagnetics) to the final polarization and the projected correlation function. The derived theoretical expressions for the polarization are tested against experimental SANS data on a soft magnetic nanocrystalline alloy.

The present paper is organized as follows: Section 2 summarizes the elementary equations of polarized neutron scattering, while Section 3 sketches the basic steps and ideas of the micromagnetic SANS theory. These two sections are well documented in the literature and may be skipped by the reader. They are included here merely to achieve a self-contained presentation. Section 4 gives the final expressions for the polarization of the scattered neutrons, discusses special sector averages and shows the results for the 2π azimuthally averaged saturated state. Section 5 furnishes the details of the polarized SANS experiment and on the investigated sample, while Section 6 presents and discusses the analysis of the experimental results. Section 7 summarizes the main findings of this study. Appendix *A* [App appa] features the expressions for the spin-resolved SANS cross sections in terms of the Fourier components of the magnetization, which enter the final expressions for the polarization, while Appendix *B* [App appb] showcases some computed examples for the polarization.

## 2. Uniaxial SANS polarization analysis

**Figure 1:**
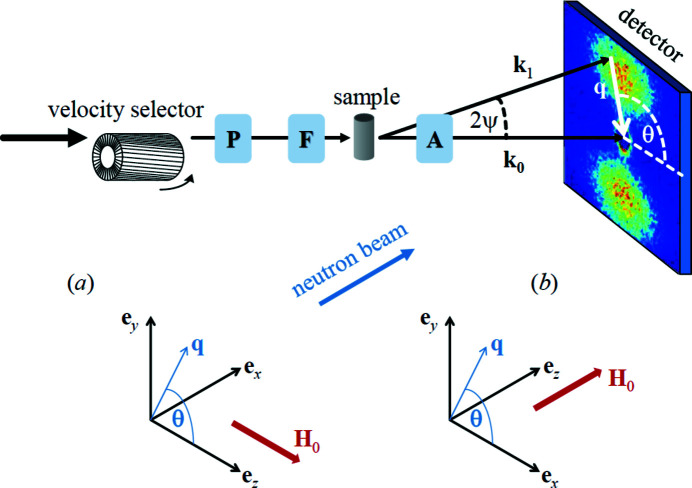
Sketch of the SANS setup and of the two most often employed scattering geometries in magnetic SANS experiments. (*a*) Applied magnetic field 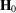 perpendicular to the incident neutron beam ( 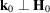 ); (*b*) 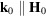 . The momentum-transfer or scattering vector 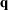 corresponds to the difference between the wavevectors of the incident ( 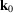 ) and the scattered ( 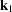 ) neutrons, *i.e.* 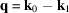 . Its magnitude for elastic scattering, 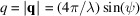 , depends on the mean wavelength λ of the neutrons and on the scattering angle 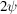 . For a given λ, sample-to-detector distance 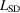 and distance 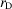 from the centre of the direct beam to a certain pixel element on the detector, the *q* value can be obtained using 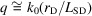 . The symbols ‘P’, ‘F’ and ‘A’ denote, respectively, the polarizer, spin flipper and analyzer, which are optional neutron optical devices. Note that a second flipper after the sample has been omitted here. In spin-resolved SANS (POLARIS) using a ^3^He spin filter, the transmission (polarization) direction of the analyzer can be switched by 180° by means of a radiofrequency pulse. SANS is usually implemented as elastic scattering ( 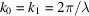 ), and the component of 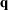 along the incident neutron beam [*i.e.* 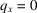 in (*a*) and 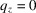 in (*b*)] is neglected. The angle θ may be conveniently used in order to describe the angular anisotropy of the recorded scattering pattern on a two-dimensional position-sensitive detector. Image taken from Michels (2021 ▸), reproduced by permission of Oxford University Press.

Fig. 1 ▸ depicts a typical uniaxial neutron polarization analysis setup. We consider the most relevant cases where the externally applied magnetic field 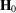 , which defines the polarization axis for both the incident and scattered neutrons, is either perpendicular or parallel to the wavevector 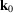 of the incident neutron beam. Note that in both scattering geometries 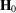 is assumed to be parallel to the 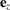 direction of a Cartesian laboratory coordinate system.

In a classical picture, the polarization 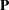 of a neutron beam containing *N* spins can be defined as the average over the individual polarizations 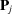 of the neutrons (Schweizer, 2006 ▸): 

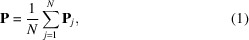

where 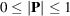 . In experimental SANS studies the beam is usually partially polarized along a certain guide-field direction (quantization axis), which we take here as the *z* direction. Assuming that the expectation values of the perpendicular polarization components vanish, *i.e.* 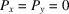 , and that 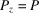 , one can then introduce the fractions 

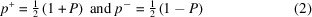

 of neutrons in the spin-up (+) and spin-down (−) states, with 

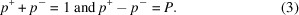

Obviously, for an unpolarized beam 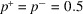 and 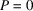 , while 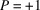 ( 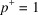 ) or 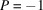 ( 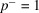 ) for a fully polarized beam.

When there is an additional analyzer behind the sample, configured such that it selects only neutrons with spins either parallel or antiparallel to the initial polarization, then one can distinguish four scattering cross sections (scattering processes) (Blume, 1963 ▸; Moon *et al.*, 1969 ▸; Schweizer, 2006 ▸): two that conserve the neutron-spin direction (++ and −−), called the non-spin-flip cross sections 

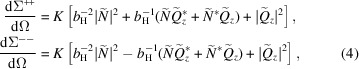

and two cross sections which reverse the neutron spin (+− and −+), called the spin-flip cross sections 

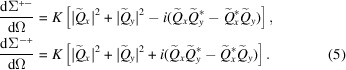

In these expressions, 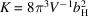 , where *V* denotes the scattering volume. 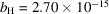 m 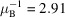 × 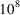 A^−1^ m^−1^ is a constant (with 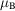 the Bohr magneton) that relates the atomic magnetic moment 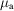 to the atomic magnetic scattering length 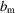 , given by (Moon *et al.*, 1969 ▸)

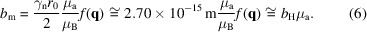




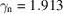
 denotes the neutron magnetic moment expressed in units of the nuclear magneton, 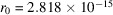 m is the classical radius of the electron and 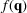 is the normalized atomic magnetic form factor, which we set to unity, 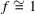 , along the forward direction. The function 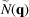 denotes the Fourier transform of the nuclear scattering-length density 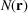 . The partial SANS cross sections, equations (4) and (5), are written here in terms of the Cartesian components of the Halpern–Johnson vector 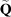 (sometimes also denoted as the magnetic interaction or magnetic scattering vector) (Halpern & Johnson, 1939 ▸): 

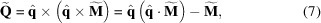

where 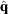 is the unit scattering vector, and 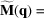 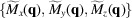 represents the Fourier transform of the magnetization vector field 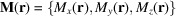 of the sample under study. The three-dimensional Fourier-transform pair of the magnetization is defined as follows: 

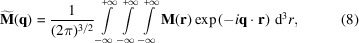




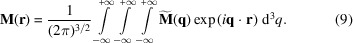

The Halpern–Johnson vector is a manifestation of the dipolar origin of magnetic neutron scattering, and it emphasizes the fact that only the components of 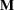 that are perpendicular to 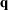 are relevant for magnetic neutron scattering. We note that different symbols for the Halpern–Johnson vector such as 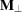 , 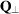 , 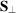 or 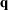 , as in the original paper by Halpern & Johnson (1939 ▸), can be found in the literature. Likewise, in many textbooks (*e.g.* Lovesey, 1984 ▸; Squires, 2012 ▸) 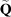 is defined with a minus sign and normalized by the factor 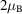 , which makes it dimensionless. 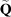 is a linear vector function of the components of 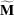 . Both 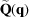 and 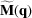 are in general complex vectors. For 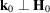 and 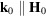 one finds, respectively (subscripts 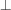 and 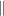 refer to the respective scattering geometry, compare Fig. 1 ▸), 

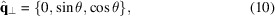




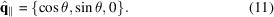

Inserting these expressions into equation (7) yields

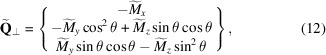




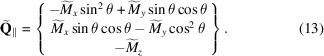

Inspection of equations (4) and (5) shows that the transverse components 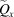 and 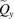 give rise to spin-flip scattering, while the longitudinal component 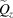 results in non-spin-flip scattering. Furthermore, if we set 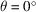 in equation (12), which corresponds to the case that the scattering vector is along the neutron polarization, we see that 

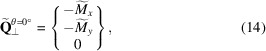

so that the magnetic scattering along the polarization direction is purely spin flip, and nuclear coherent and magnetic scattering are fully separated in the perpendicular scattering geometry [compare also with equations (4) and (5) for the non-spin-flip and the spin-flip SANS cross sections and with equation (21) for the final polarization]. In the case 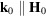 [equation (13)], spin-flip scattering probes only the transverse magnetization Fourier components 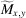 , whereas the longitudinal scattering is entirely contained in the non-spin-flip channel, in contrast to the 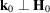 geometry. We emphasize that nuclear-spin-dependent SANS is not taken into account in this paper, so that the corresponding scattering contributions do not show up in equations (4) and (5).

The total SANS cross section 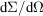 can be expressed in terms of the initial spin populations 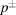 as (Blume, 1963 ▸; Moon *et al.*, 1969 ▸; Schweizer, 2006 ▸)

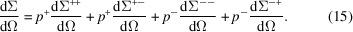

Inserting the above expressions for 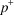 and 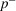 [equations (2) and (3)] and for the partial SANS cross sections 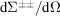 and 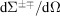 [equations (4) and (5)], equation (15) evaluates to

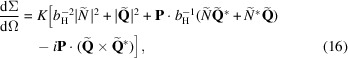

which, using 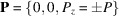 , can be rewritten as

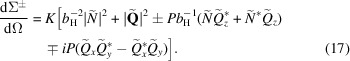

Since the cross section is a scalar quantity and the polarization is an axial vector (or pseudovector), equation (16) shows that the system under study must itself contain an axial vector. As emphasized by Maleev (2002 ▸), examples for such built-in pseudovectors are related to the interaction of a polycrystalline sample with an external magnetic field (inducing an average magnetization directed along the applied field), the existence of a spontaneous magnetization in a ferromagnetic single crystal, the antisymmetric Dzyaloshinskii–Moriya interaction (DMI), mechanical (torsional) deformation or the presence of spin spirals. If on the other hand there is no preferred axis in the system, then 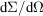 is independent of 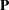 . Examples include a collection of randomly oriented non­interacting nuclear (electronic) spins, which describe the general case of nuclear (paramagnetic) scattering at not-too-low temperatures and large applied fields, or a multi-domain ferromagnet with a random distribution of the domains. The same condition – existence of an axial system vector – applies for neutrons to be polarized in the scattering process [compare the last two terms in equation (21) below].

In the domain of magnetic SANS it is customary to denote experiments with a polarized incident beam only, and no spin analysis of the scattered neutrons, with the acronym SANSPOL. The two SANSPOL cross sections 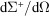 and 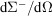 (also sometimes denoted as the half-polarized SANS cross sections) combine non-spin-flip and spin-flip scattering contributions, according to ( 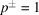 )

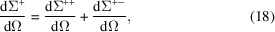




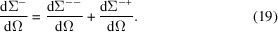

Finally, noting that an unpolarized beam can be viewed as consisting of 50% spin-up and 50% spin-down neutrons [compare equations (2) and (3)], the unpolarized SANS cross section is obtained as [compare equation (15)] 

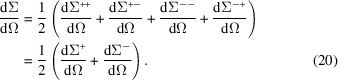

The polarization 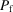 of the scattered beam along the direction of the incident neutron polarization *P* is obtained from the following relation (Blume, 1963 ▸; Moon *et al.*, 1969 ▸; Schweizer, 2006 ▸): 

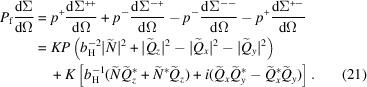

The first four terms in the second line on the right-hand side of equation (21) demonstrate that nuclear scattering (to be more precise, the nuclear coherent scattering, the isotopic disorder scattering, and 1/3 of the nuclear-spin-dependent scattering) and scattering due to the longitudinal component 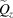 of the magnetic scattering vector 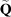 do not reverse the initial polarization, while the two transverse components 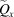 and 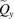 give rise to spin-flip scattering. The last two terms in equation (21) do create polarization: these are the familiar nuclear–magnetic interference term ( 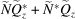 ), which is commonly used to polarize beams, and the chiral term 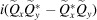 , which is of relevance in inelastic scattering (dynamic chirality) (Okorokov *et al.*, 1981 ▸; Maleev, 2002 ▸; Grigoriev *et al.*, 2015 ▸), in elastic scattering on spiral structures and weak ferromagnets (canted antiferromagnets) (Thoma *et al.*, 2021 ▸), or in the presence of the DMI in microstructural-defect-rich magnets (Michels *et al.*, 2019 ▸; Quan *et al.*, 2020 ▸). We remind the reader that nuclear-spin-dependent scattering is not taken into account in the expressions for the magnetic SANS cross sections. In the general expression for the polarization of the scattered neutrons, a term 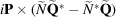 appears (Schweizer, 2006 ▸), which is ignored in equation (21). This term rotates the polarization perpendicular to the initial polarization and cannot be observed in the uniaxial setup. We emphasize that in linear neutron polarimetry it is not possible to distinguish between a rotation of the polarization vector and a change of its length (Moon *et al.*, 1969 ▸; Maleev, 2002 ▸).

From equation (21) it follows that the polarization 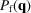 of the scattered neutron beam at momentum-transfer vector 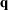 can be expressed as (Maleev *et al.*, 1963 ▸; Blume, 1963 ▸; Brown, 2006 ▸)

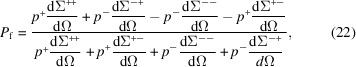

which for 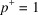 ( 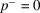 ) and 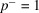 ( 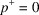 ) reduces to, respectively, 

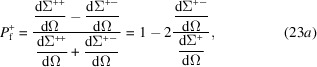




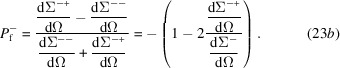

Note that, for the following analysis, we drop the minus sign in front of the round brackets in equation (23*b* [Disp-formula fd23b]). For a quantitative analysis of 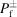 , a theoretical model for the magnetization Fourier components 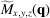 and for 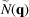 is required. This will be discussed in the next section.

## 3. Sketch of micromagnetic SANS theory

Michels *et al.* (2016 ▸) presented a theory for the magnetic SANS cross section of bulk ferromagnets. The approach, which considers the response of the magnetization to spatially inhomogeneous magnetic anisotropy fields and magnetostatic fields, is based on the continuum theory of micromagnetics, valid close to magnetic saturation, and takes the antisymmetric DMI into account. Here, we recall the basic steps and ideas. The starting point is the static equations of micromagnetics for the bulk, which can be derived from the following expression for the magnetic Gibbs free energy (Brown, 1963 ▸; Aharoni, 2000 ▸; Kronmüller & Fähnle, 2003 ▸): 

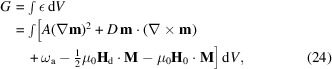

where the first term denotes the energy due to the isotropic exchange interaction, the second term is the antisymmetric Dzyaloshinskii–Moriya energy (assuming a cubic symmetry), 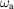 is the anisotropy energy density, and the last two terms are the energies related, respectively, to the dipolar interaction and the externally applied magnetic field; 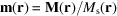 (Michels *et al.*, 2016 ▸). Variational calculus then leads to the following balance-of-torques equation: 

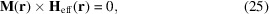

which expresses the fact that at static equilibrium the torque on the magnetization 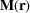 due to an effective magnetic field 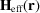 vanishes everywhere inside the material. The effective field is defined as the functional derivative of the ferromagnet’s total energy-density functional ε with respect to the magnetization: 

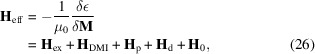

where 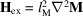 is the exchange field, 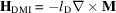 is due to the DMI, 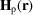 is the magnetic anisotropy field, 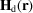 is the magnetostatic field and 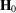 is a uniform applied magnetic field; 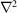 is the Laplace operator and 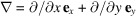 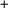 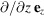 is the gradient operator, where the unit vectors along the Cartesian laboratory axes are, respectively, denoted by 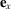 , 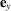 and 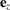 ( 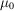 , vacuum permeability). The micromagnetic length scales 

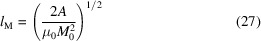

and 

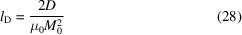

are, respectively, related to the magnetostatic interaction and to the DMI. The values for the DMI constant *D* and for the exchange-stiffness constant *A* are assumed to be uniform throughout the material, in contrast to the local saturation magnetization 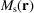 , which is assumed to depend explicitly on the position 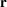 [see also Metlov & Michels (2015 ▸)]; 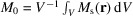 denotes the macroscopic saturation magnetization of the sample, which can be measured with a magnetometer. Typical values for the above length scales are 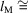 5–10 nm (Kronmüller & Fähnle, 2003 ▸) and 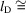 1–2 nm. However, due to the lack of an established database for *D* values, this estimate should be considered with some care.

The solution of the linearized version of equation (25) [see Michels *et al.* (2016 ▸) for details] provides closed-form expressions for the transverse Fourier components 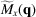 and 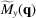 . Together with theoretical models (or even experimental data) for the longitudinal magnetization Fourier component 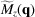 and for the nuclear scattering amplitude 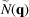 , these analytical solutions can be employed to compute the corresponding terms in the SANS cross sections (see Appendix *A* [App appa]) and, hence, to evaluate the final polarizations. Averaging over the directions of the magnetic anisotropy field in the plane perpendicular to the applied field, the magnetic terms for the transverse magnetic field geometry ( 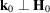 , 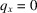 ) are

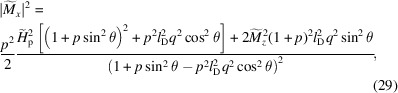




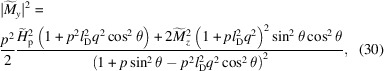




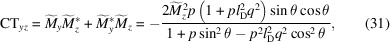




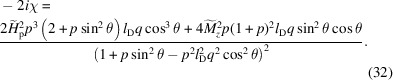

The results for the parallel field geometry ( 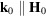 , 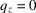 ) are

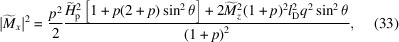




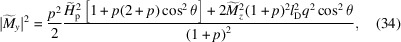




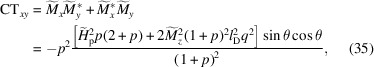




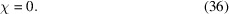

In equations (29)–(36), 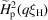 denotes the magnitude-square of the Fourier transform of the magnetic anisotropy field, and 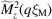 is the Fourier component of the longitudinal magnetization. We also emphasize that 

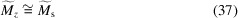

is assumed in the approach-to-saturation regime, which Bersweiler *et al.* (2022 ▸) defined for applied fields where the reduced magnetization 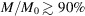 . These functions characterize the strength and spatial structure of, respectively, the magnetic anisotropy field 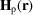 , with correlation length 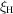 , and the local saturation magnetization 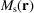 , with correlation length 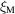 . 

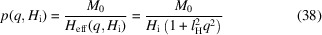

is a dimensionless function, where 

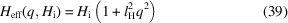

is the effective magnetic field [not to be confused with 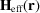 in equation (25)], which depends on the internal magnetic field 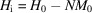 (*N*, demagnetizing factor), on 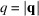 and on the exchange length of the field 

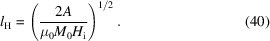

The latter quantity is a measure for the size of inhomogeneously magnetized regions around microstructural lattice defects (Mettus & Michels, 2015 ▸). The Fourier coefficient of the longitudinal magnetization 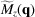 provides information on the spatial variation of the saturation magnetization 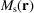 ; for instance, 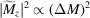 in a multiphase magnetic nanocomposite, where 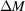 denotes the jump of the magnetization magnitude at internal (particle–matrix) interfaces (Honecker & Michels, 2013 ▸). Moreover, while the squared Fourier components and the cross terms are even functions of 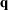 , it is easily seen that the chiral term 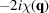 [equation (32)] is asymmetric in 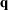 , which is due to the DMI term: at small fields, when the term 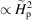 in the numerator of equation (32) dominates, two extrema parallel and antiparallel to the field axis are observed, whereas at larger fields, when the term 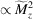 dominates, additional maxima and minima appear approximately along the detector diagonals (Michels *et al.*, 2016 ▸).

By inserting equations (29)–(36) into the SANS cross sections (see Appendix *A* [App appa]) and by specifying particular models for the nuclear scattering function 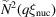 , the longitudinal magnetic Fourier component 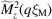 and the Fourier coefficient of the magnetic anisotropy field 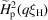 , one obtains 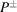 as a function of the magnitude and orientation of the scattering vector 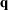 , the applied magnetic field 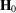 , the magnetic interaction parameters (*A*, *D*, 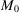 , 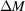 , 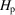 , 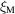 , 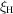 ) and microstructural quantities (particle-size distribution, crystallographic texture *etc*.). We emphasize that the expressions (29)–(36) for the Fourier components can of course also be employed directly in the SANS cross sections to analyze experimental scattering data [see *e.g.* Bersweiler *et al.* (2022 ▸) for a recent example]. Later on in the paper, for graphically displaying 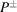 , we have assumed that 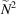 , 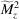 and 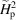 are all isotropic (*i.e.* θ independent), as is appropriate for polycrystalline texture-free bulk ferromagnets, and that they can be represented by Lorentzian-squared functions, *i.e.*


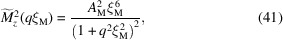




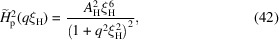




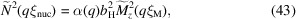

where the amplitudes 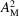 and 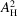 (both in units of A^2^ nm^−2^) are, respectively, related to the mean-square magnetization fluctuation and anisotropy-field variation. Of course, other scattering functions such as the form factor of a sphere and various structure-factor models (*e.g.* a Percus–Yevick hard-sphere structure factor) can be straightforwardly implemented (Mettus & Michels, 2015 ▸). The characteristic structure sizes of 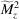 and 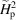 are generally different. We remind the reader that these are related, respectively, to the spatial extent of regions with uniform saturation magnetization ( 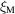 ) and magnetic anisotropy field ( 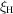 ). Measurement of the magnetic-field-dependent Guinier radius provides a means to determine these correlation lengths as well as the exchange-stiffness constant *A* (Michels *et al.*, 2020 ▸). A simple example where 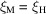 is a collection of homogeneous and defect-free magnetic nanoparticles in a magnetic and homogeneous matrix. If, on the other hand, atomically sharp grain boundaries are introduced into such particles, then the direction of the magnetic anisotropy field changes due to the changing set of crystallographic directions at the intraparticle interfaces, but the value of 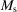 may remain the same, so that 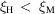 . Honecker & Michels (2013 ▸) showed, assuming 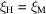 and using the sphere form factor for both 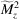 and 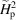 , that it is the ratio of 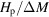 (related to the amplitudes 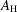 and 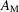 ) that determines the angular anisotropy and the asymptotic power-law dependence of 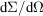 as well as the characteristic decay length of spin-misalignment fluctuations. The ratio of nuclear to longitudinal magnetic scattering is denoted by α, which for the general case (that the nuclear correlation length 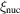 is different from 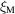 ) is a function of *q*. Here, we do not specify a particular 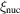 and assume this characteristic size to be contained in 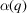 .

## 4. Polarization of scattered beam

When the expressions for the elastic differential spin-flip and SANSPOL cross sections 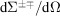 and 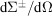 [equations (66)–(70)] are inserted into equations (23*a* [Disp-formula fd23a]) and (23*b* [Disp-formula fd23b]), and use is made of the expressions for the magnetization Fourier components [equations (29)–(36)], one obtains, respectively, for the transverse and longitudinal scattering geometry

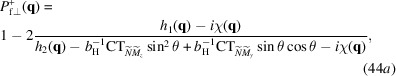




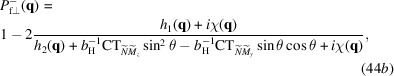




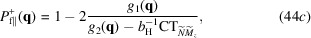




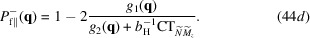

The functions 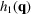 , 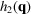 , 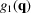 and 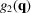 are independent of the incident neutron beam polarization and are defined as

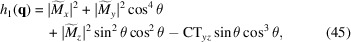




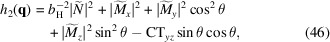




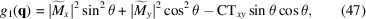




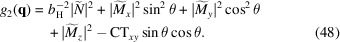

At complete magnetic saturation, when 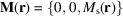 , these expressions reduce to

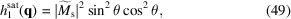




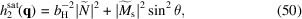




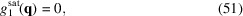




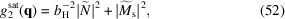

where 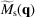 is the Fourier transform of 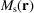 . As can be seen from equations (44*a* [Disp-formula fd44a]) and (44*b* [Disp-formula fd44b]), the difference between 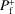 and 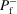 resides, for 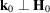 , in the nuclear–magnetic interference terms 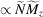 and 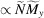 , and in 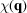 , while for 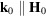 the two polarizations differ only by the term 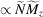 [equations (44*c* [Disp-formula fd44c]) and (44*d* [Disp-formula fd44d])]. We also remind the reader that the Fourier coefficients in the above expressions are evaluated in the plane of the detector, which for the perpendicular scattering geometry corresponds to the plane 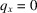 and for the parallel geometry to the plane 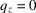 (compare Fig. 1 ▸).

The 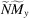 contribution to equations (44*a* [Disp-formula fd44a]) and (44*b* [Disp-formula fd44b]) requires special consideration. This term is expected to be negligible for a polycrystalline statistically isotropic ferromagnet with vanishing fluctuations of the saturation magnetization. This can be seen by scrutinizing the following expression for the 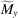 magnetization Fourier component in the perpendicular scattering geometry, corresponding to the plane 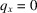 (Michels, 2021 ▸): 

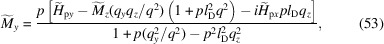

where 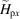 and 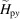 denote the Cartesian components of the Fourier transform of the magnetic anisotropy field. If we assume that the nuclear scattering is isotropic and that 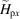 and 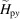 vary randomly in the plane perpendicular to the field, then the corresponding averages over the direction of the anisotropy field vanish. The only remaining term in the 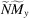 contribution is then ( 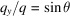 ; 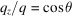 )

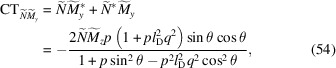

where we have furthermore assumed that both 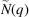 and 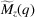 are real valued, as is done throughout this paper. Note that equation (54) still needs to be multiplied with the term 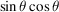 in order to obtain the corresponding contribution to 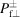 [compare equations (44*a* [Disp-formula fd44a]) and (44*b* [Disp-formula fd44b])]. For homogeneous single-phase materials with 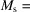 constant, the 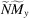 contribution is expected to be negligible, and we are not aware that this has been reported experimentally. However, for materials exhibiting strong spatial nanoscale variations in the saturation magnetization, *i.e.* 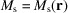 , such as magnetic nanocomposites or porous ferromagnets, it should be possible to observe this scattering contribution in polarized SANS experiments.

### 4.1. Sector averages

Carrying out a 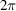 azimuthal average of 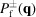 , which are maps with numbers varying between 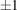 , may result in a significant loss of information (compare *e.g.* Figs. 10 and 11 below). It is therefore often advantageous to consider cuts of 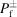 along certain directions in 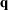 space. This might also be of relevance for other spin-manipulating techniques such as SEMSANS, which is a one-dimensional technique that only measures correlations in the encoding direction (Li *et al.*, 2021 ▸). However, one has to keep in mind that SEMSANS is a real-space technique that (similar to SESANS and DFI) essentially measures the cosine Fourier transform of the cross section. Nevertheless, the analytical expressions for the magnetization Fourier components can be used in such a transform to obtain information on the magnetic interactions via the projected correlation function.

Sector averages are straightforwardly obtained by evaluating equations (44*a* [Disp-formula fd44a])–(44*d* [Disp-formula fd44d]) [using equations (45)–(52)] for certain angles θ. For instance, for the perpendicular scattering geometry and for 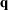 along the vertical direction on the detector ( 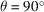 ), we obtain [ 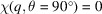 , compare equation (32)]

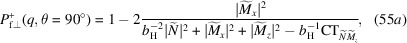




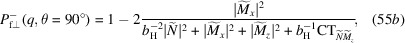

where [compare equation (29)] 

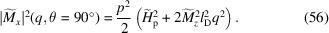

At saturation ( 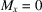 ), 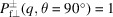 , except for the case 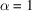 , where 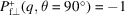 . We also see that information on the DMI is contained in 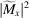 via the length scale 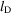 . For 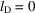 , 
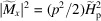
 .

For the perpendicular scattering geometry and 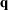 along the horizontal direction ( 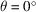 ), we obtain

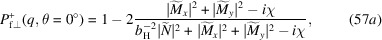




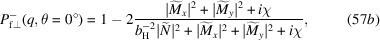

where [compare equations (29) and (30)] 

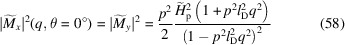

and [compare equation (32)] 

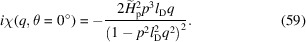

At saturation ( 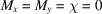 ) and for nonzero nuclear scattering, 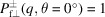 . For 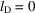 , 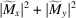 = 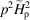 and 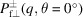 contains information on the transverse spin components.

### 4.2. Saturated state

At saturation and for 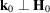 , it is readily verified from equations (44*a* [Disp-formula fd44a]) and (44*b* [Disp-formula fd44b]) using equations (49)–(52), 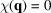 and 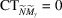 that 

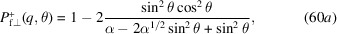




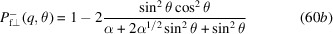

depend exclusively on the ratio 

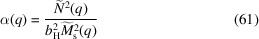

of nuclear to longitudinal magnetic scattering [compare equation (43)]. The possible angular anisotropy of α is not considered in this paper. Since for 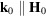 the spin-flip SANS cross section vanishes at saturation ( 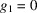 ), we see that 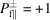 . The azimuthally averaged [ 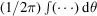 ] versions of equations (60*a* [Disp-formula fd60a]) and (60*b* [Disp-formula fd60b]) read

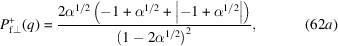




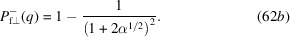

The function 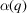 can be a monotonically increasing or decreasing function of *q*, and it can even exhibit local extrema. In the following, we will consider the cases of 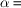 constant and 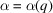 using the experimental data of the soft magnetic Fe-based alloy NANOPERM (Michels *et al.*, 2012 ▸).

#### 4.2.1. α = constant

**Figure 2:**
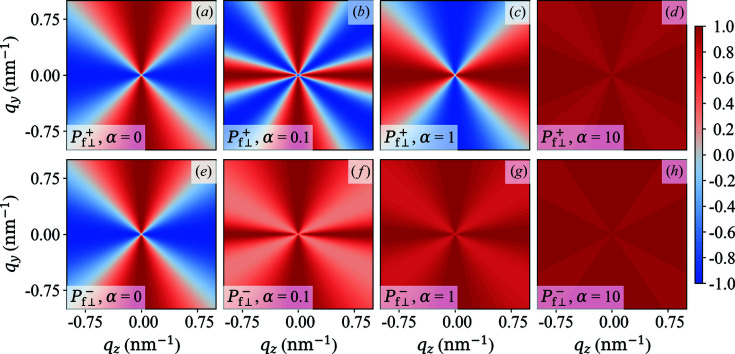
Plot of 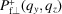 (upper row) and 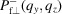 (lower row) in the saturated state for different values of α (see insets) [equations (60*a*)[Disp-formula fd60a] and (60*b*)[Disp-formula fd60b]].

**Figure 3:**
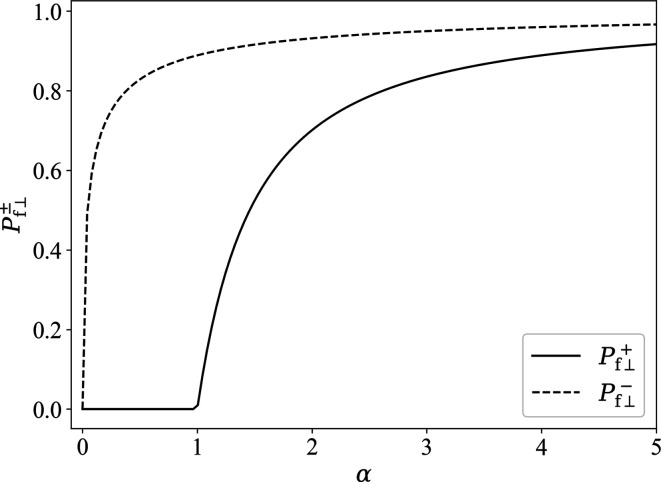
Plot of 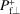 and 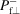 (see inset) in the saturated state as a function of α [equations (62*a*)[Disp-formula fd62a] and (62*b*)[Disp-formula fd62b]].

Fig. 2 ▸ displays the two-dimensional polarization 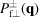 of the scattered neutrons in the saturated state as a function of α = constant. The case of constant α is very rarely realized in experimental situations, and we consider it here only as a starting point for our discussion and for the comparison with the experimentally more relevant situation of 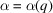 . For 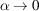 it follows that 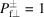 − 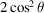 [Figs. 2 ▸(*a*) and 2 ▸(*e*)], while 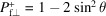 [Fig. 2 ▸(*c*)] and 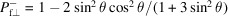 [Fig. 2 ▸(*g*)] for 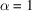 . When nuclear coherent scattering is dominating ( 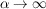 ), we see that 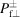 both tend to unity, as expected. The corresponding 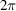 azimuthally averaged functions [equations (62*a* [Disp-formula fd62a]) and (62*b* [Disp-formula fd62b])] are plotted in Fig. 3 ▸. One readily verifies that 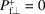 for 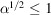 , which further underlines the loss of information when a 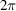 azimuthal average is carried out [compare *e.g.* Fig. 2 ▸(*b*)].

#### 4.2.2. α = α(*q*)

**Figure 4:**
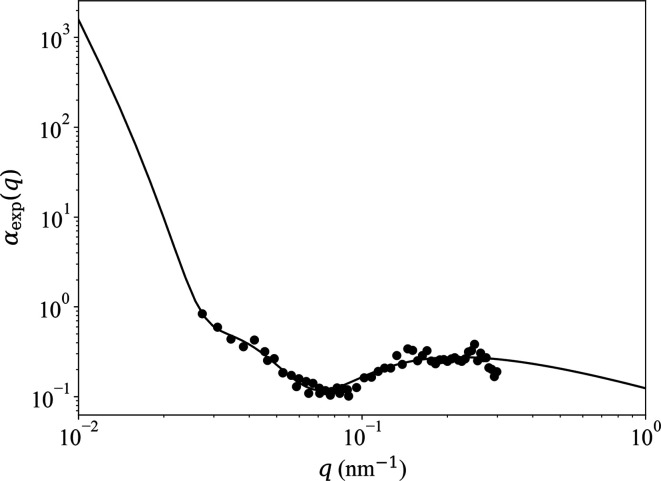
Black circles: experimental ratio 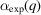 of nuclear to magnetic scattering of the two-phase alloy NANOPERM (Michels *et al.*, 2012 ▸) ( 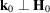 ; 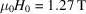 ; log–log plot). Solid line: power-law fit to parametrize the experimental data [equation (63)]. The fit has been restricted to the interval 0.03 < *q* < 0.3 nm^−1^, but the fit function is displayed for 0.01 < *q* < 1.0 nm^−1^.

**Figure 5:**
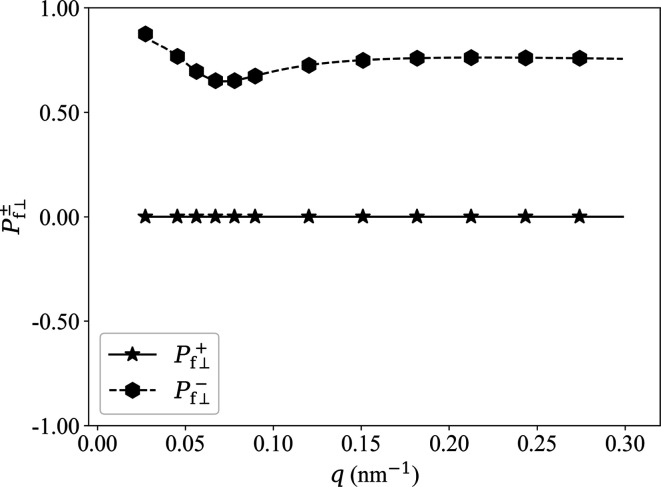
Plot of 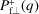 (solid line) and 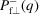 (dashed line) of NANOPERM using equations (62*a*)[Disp-formula fd62a] and (62*b*)[Disp-formula fd62b] with 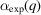 given by equation (63) and 0.03 < *q* < 0.3 nm^−1^.

Fig. 4 ▸ shows the experimentally determined ratio 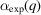 (Michels *et al.*, 2012 ▸) of nuclear to magnetic scattering of the two-phase alloy NANOPERM. Within the experimental *q* range of 0.03 < *q* < 0.3 nm^−1^, these data for 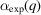 have been fitted by a power law in 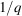 to obtain the functions 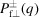 , which are depicted in Fig. 5 ▸. The used fit function for 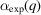 is

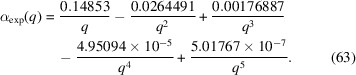

This expression will be used in the analysis of the experimental data (see Section 6).

### 4.3. Nonsaturated state

Appendix *B* [App appb] features some theoretical results for 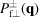 for various combinations of the magnetic interaction parameters [applied magnetic field, ratio of 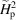 to 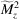 , 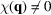 ]. For a statistically isotropic ferromagnet, the two-dimensional distribution of the polarization of the scattered neutrons is isotropic (θ independent) for the longitudinal scattering geometry ( 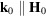 ), as are the corresponding SANS cross sections. This is in contrast to the 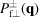 for the transverse geometry ( 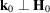 ), which are highly anisotropic. In the following, we will use the theoretical expressions for 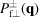 to analyze experimental data on the soft magnetic two-phase nanocrystalline alloy NANOPERM.

## 5. Experimental details

The polarized neutron experiment was carried out at room temperature at the instrument D22 at the Institut Laue–Langevin, Grenoble, France. Incident neutrons with a mean wavelength of λ = 8 Å and a wavelength broadening of 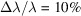 (FWHM) were selected by means of a velocity selector. The beam was polarized using a 1.2 m-long remanent Fe–Si supermirror transmission polarizer (*m* = 3.6), which was installed immediately after the velocity selector. A radio­frequency spin flipper, installed close to the sample position, allowed us to reverse the initial neutron polarization. The external magnetic field (provided by an electromagnet) was applied perpendicular to the wavevector 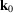 of the incident neutrons (compare Fig. 1 ▸). Measurement of the four partial POLARIS cross sections 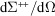 , 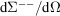 , 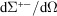 and 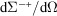 was accomplished through a polarized ^3^He spin-filter cell, which was installed inside the detector housing, about 1 m away from the sample position. The polarization between polarizer, radiofrequency flipper and ^3^He filter was maintained by means of magnetic guide fields of the order of 1 mT. The efficiencies of the polarizer, spin flipper and ^3^He analyzer were, respectively, 90, 99 and 87.5%. The scattered neutrons were detected by a multitube detector which consists of 128 × 128 pixels with a resolution of 8 × 8 mm. Neutron data reduction, including corrections for background scattering and spin leakage (Wildes, 2006 ▸), was performed using the *GRASP* (Dewhurst, 2021 ▸) and *BerSANS* (Keiderling, 2002 ▸; Keiderling *et al.*, 2008 ▸) software packages.

The sample under study was a two-phase magnetic nanocomposite from the NANOPERM family of alloys (Suzuki & Herzer, 2006 ▸) with a nominal composition of (Fe_0.985_Co_0.015_)_90_Zr_7_B_3_ (Suzuki *et al.*, 1994 ▸; Ito *et al.*, 2007 ▸). The alloy was prepared by melt spinning, followed by a subsequent annealing treatment for 1 h at 883 K, which resulted in the precipitation of body-centered cubic iron nanoparticles in an amorphous magnetic matrix. The average iron particle size of *D* = 15 ± 2 nm was determined by the analysis of wide-angle X-ray diffraction data. The crystalline particle volume fraction is about 65% and the saturation magnetization of the alloy amounts to 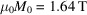 . The exchange-stiffness constant 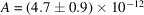 J m^−1^ has previously been determined by the analysis of the field-dependent unpolarized SANS cross section (Honecker *et al.*, 2013 ▸). For the SANS experiments, several circular discs with a diameter of 10 mm and a thickness of about 20 µm were stacked and mounted on a Cd aperture [for further details see Michels *et al.* (2012 ▸) and Honecker *et al.* (2013 ▸)].

## 6. Experimental results and discussion

**Figure 6:**
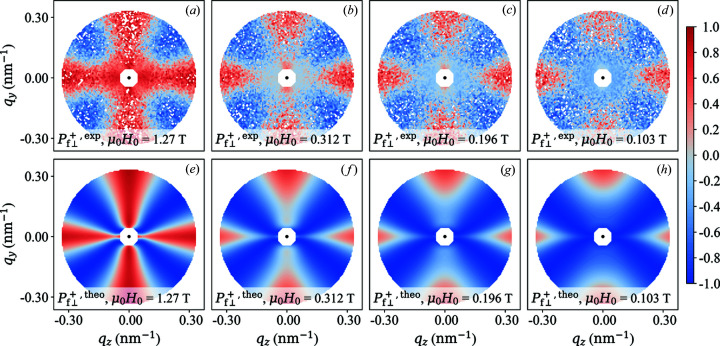
Qualitative comparison between experiment and theory. (*a*)–(*d*) Two-dimensional experimental polarization 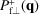 of the scattered neutrons of NANOPERM [(Fe_0.985_Co_0.015_)_90_Zr_7_B_3_] at a series of applied magnetic fields (see insets). 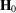 is horizontal in the plane. The range of momentum transfers is restricted to 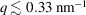 . (*e*)–(*h*) Prediction by the analytical micromagnetic theory (no free parameters) using the experimental ratio 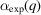 [equation (63)] and the structural ( 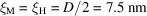 ) and magnetic ( 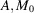 ) interaction parameters of NANOPERM [see text, Michels *et al.* (2012 ▸) and Honecker *et al.* (2013 ▸)]. The central white octagons mark the position of the beamstop.

**Figure 7:**
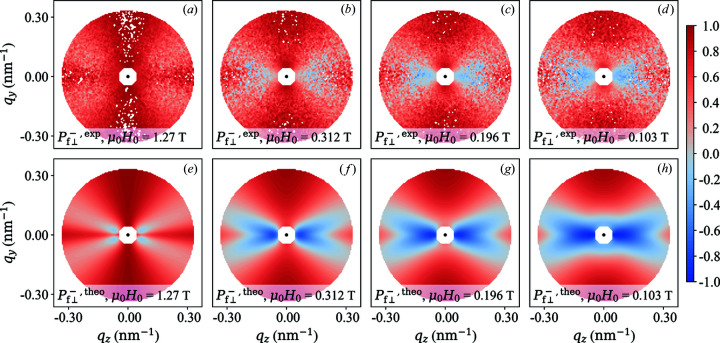
Similar to Fig. 6 ▸, but for 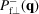 .

The two-dimensional experimental distribution of the polarization of NANOPERM is depicted in Figs. 6 ▸ ( 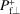 ) and 7 ▸ ( 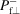 ) at selected field values together with a qualitative comparison with the simulated polarization based on the micromagnetic SANS theory [see Michels *et al.* (2012 ▸) for some selected spin-resolved SANS cross sections]. The theory uses as input values the experimental ratio 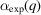 [equation (63)] and the structural ( 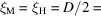 7.5 nm) and magnetic ( 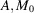 ) interaction parameters. In agreement with the previous micromagnetic SANS data analysis of this sample (Michels *et al.*, 2012 ▸; Honecker *et al.*, 2013 ▸), we have set the ratio 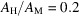 . We also assumed that both spin-flip channels are equal, *i.e.* 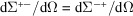 , a constraint that was already imposed during the spin-leakage correction. The overall qualitative agreement between experiment and theory (no free parameters) is evident, although the angular anisotropy of the data does not exhibit a large variation with field. Only at the smallest momentum transfers can one notice a change in the anisotropy with decreasing field (in particular in 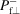 ), which is related to the emerging spin-misalignment scattering; compare *e.g.* scattering terms 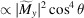 and 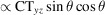 in equations (66) and (69). We also note the existence of (seemingly isotropic) scattering contributions at small 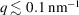 (especially at 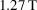 ), which are probably due to large-scale structures that are not contained in the micromagnetic theory [compare Figs. 6 ▸(*a*) and 6 ▸(*e*) and Figs. 7 ▸(*a*) and 7 ▸(*e*)].

**Figure 8:**
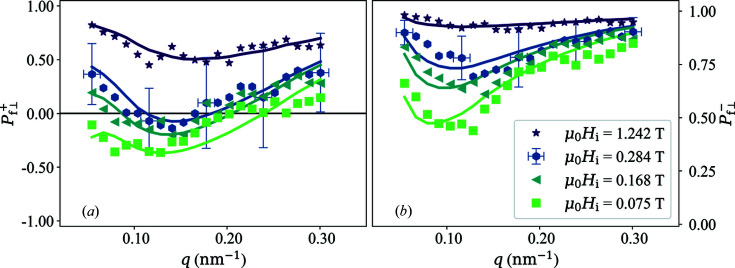
(Data points) Experimental polarizations 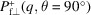 (*a*) and 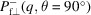 (*b*) of the scattered neutrons of NANOPERM [(Fe_0.985_Co_0.015_)_90_Zr_7_B_3_] at a series of internal magnetic fields (see inset). For clarity of presentation, error bars are only shown for one field. (Solid lines) Prediction by the analytical micromagnetic theory [equations (44*a*)[Disp-formula fd44a] and (44*b*)[Disp-formula fd44b]] using the ratio 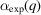 [equation (63)]. Note the different scales on the ordinates in (*a*) and (*b*).

Due to the relatively large statistical noise in the two-dimensional 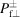 maps we did not fit the experimental data directly to the theoretical expressions. Therefore, in the following, we consider one-dimensional experimental polarization data, which were obtained by averaging the two-dimensional polarized SANS cross sections over 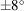 along the vertical direction ( 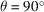 ). These averages were used in equations (23*a* [Disp-formula fd23a]) and (23*b* [Disp-formula fd23b]) to obtain 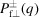 . The resulting data in Fig. 8 ▸ were then fitted using the general equations (44*a* [Disp-formula fd44a]) and (44*b* [Disp-formula fd44b]) (also averaged over 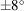 along 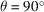 ). Adjustable parameters are the amplitudes (scaling parameters) 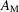 and 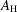 of, respectively, 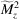 and 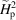 as well as the corresponding correlation lengths 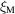 and 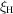 [compare equations (41) and (42)]. The field-dependent micromagnetic exchange length 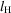 , which is contained in the dimensionless function 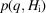 [compare equations (38)–(40)], is computed at each field using the materials parameters *A* and 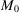 ; *A* is treated here as an additional adjustable parameter. For 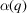 we used equation (63), and the DMI has been ignored in the data analysis ( 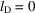 ). Since the 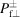 differ only by the 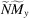 and 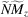 interference terms, we have fitted the 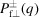 data corresponding to the same field simultaneously. The applied field 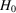 has been corrected for demagnetizing effects.

**Figure 9:**
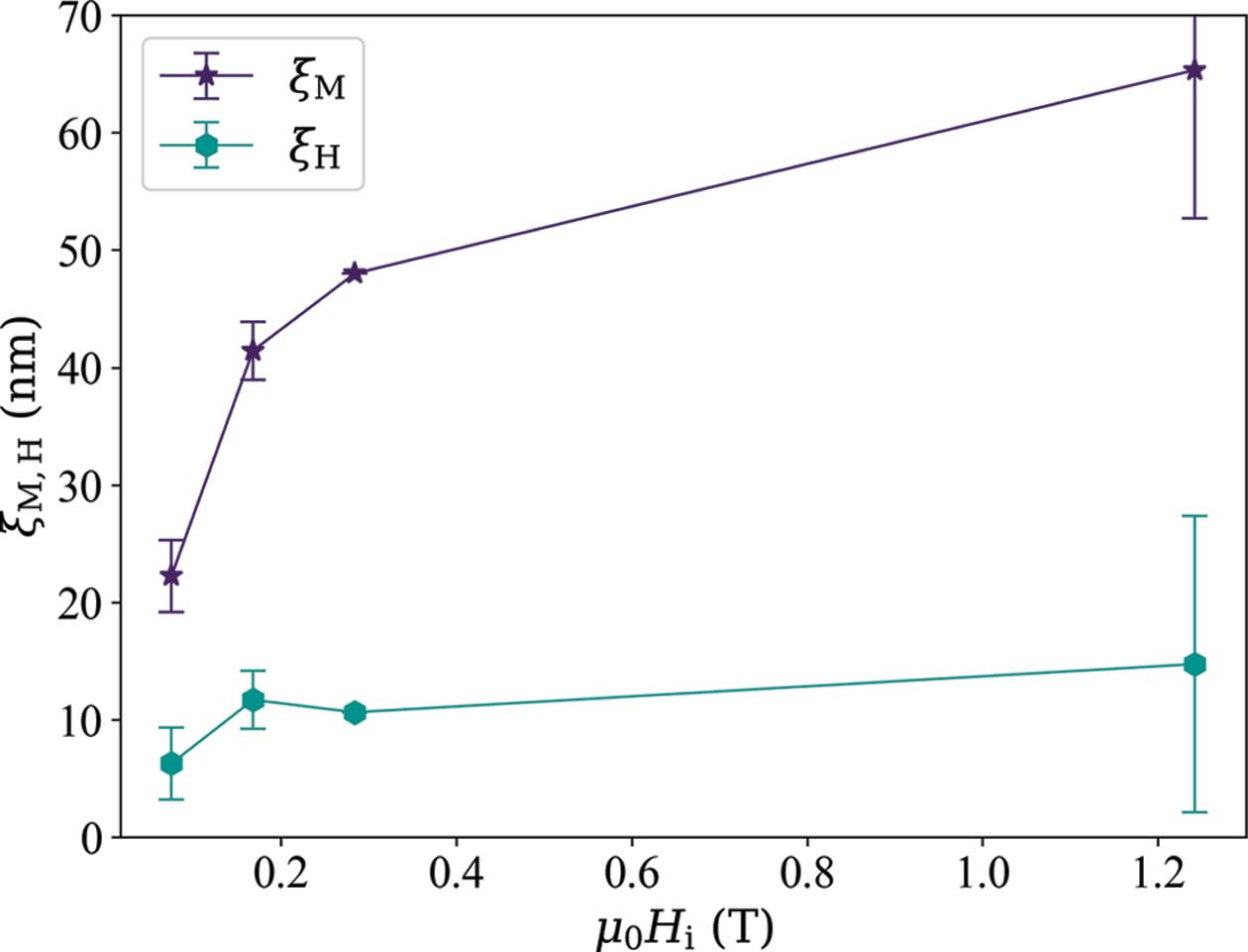
Resulting best-fit values for the correlation lengths 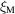 and 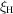 (see inset). Lines are a guide to the eye.

**Figure 10:**
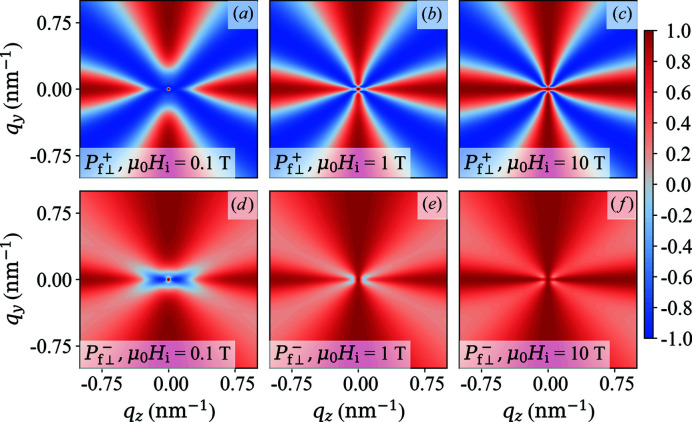
Plot of 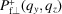 (upper row) and 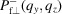 (lower row) for different applied magnetic fields 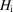 (see insets). 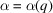 [equation (63)], 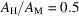 , 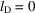 .

**Figure 11:**
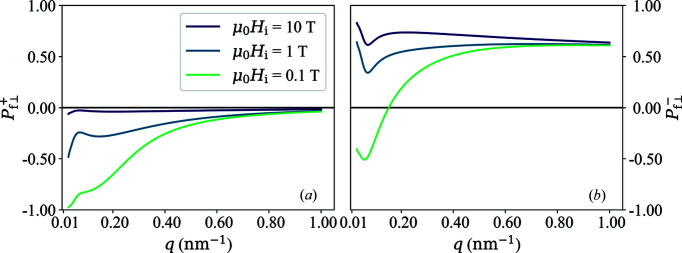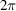 azimuthally averaged 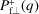 (*a*) and 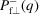 (*b*) of the data shown in Fig. 10 ▸.

The fits in Fig. 8 ▸ (solid lines) provide a reasonable description of the experimental data. The obtained values for 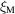 and 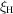 are shown in Fig. 9 ▸; 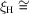 6–15 nm is at all fields consistently of the order of the particle size, while 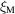 takes on larger values between about 22 and 65 nm. For the exchange-stiffness constant, we obtain (from the four local fits) best-fit values in the range *A* = (4.8–9.7) × 10^−12^ J m^−1^. These values agree very well with data in the literature (Honecker *et al.*, 2013 ▸; Bersweiler *et al.*, 2022 ▸).

Clearly, more experiments are needed in order to further scrutinize the predictions of the present micromagnetic theory for the uniaxial polarization analysis of bulk ferromagnets. In this respect, the development of computational tools to directly analyze the two-dimensional polarization maps using different form-factor and structure-factor expressions for 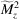 and 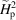 , and possibly the inclusion of a particle-size distribution function, would be desirable. Likewise, SANS measurements at a preferably saturating magnetic field are necessary to determine the nuclear SANS cross section, *e.g.* via a horizontal average of the non-spin-flip SANS cross section.

## 7. Summary and conclusions

We have provided a micromagnetic theory for the uniaxial polarization of the scattered neutrons of bulk ferromagnets, as it can be measured by means of the small-angle neutron scattering (SANS) method. The theoretical expressions contain the effects of an isotropic exchange interaction, the Dzyaloshinskii–Moriya interaction, magnetic anisotropy, magnetodipolar interaction and an external magnetic field. The theory has been employed to analyze experimental data on a soft magnetic nanocrystalline alloy; it may provide information on the magnetic interactions (exchange and DMI constants) and on the spatial structures of the magnetic anisotropy and magnetostatic fields. Given that uniaxial polarization analysis is becoming more and more available on SANS instruments worldwide and in view of the recent seminal progress made regarding several techniques which exploit the neutron polarization degree of freedom to characterize large-scale magnetic structures (SESANS, DFI, SEMSANS), we believe that the results of this paper open up a new avenue for magnetic neutron data analysis on mesoscopic magnetic systems. This is because the presented micromagnetic SANS framework forms the basis for all of these new and promising polarization encoding techniques, with the paper by Rekveldt *et al.* (2006 ▸) providing the relevant expressions that link the magnetization distribution to the final polarization and the projected correlation function.

## Figures and Tables

**Figure 12:**
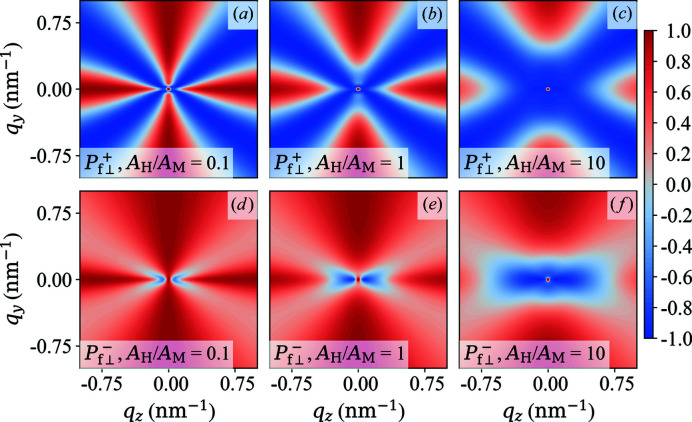
Plot of 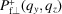 (upper row) and 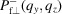 (lower row) for different ratios of 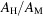 (see insets). 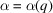 [equation (63)], 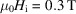 , 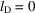 .

**Figure 13:**
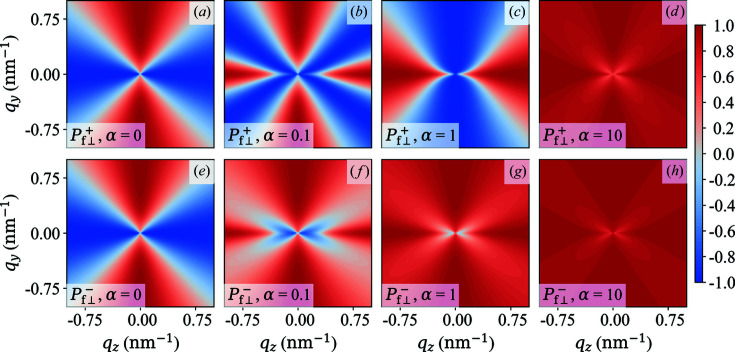
Plot of 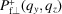 (upper row) and 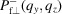 (lower row) for different values of 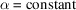 (see insets). 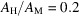 , 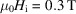 , 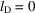 .

**Figure 14:**
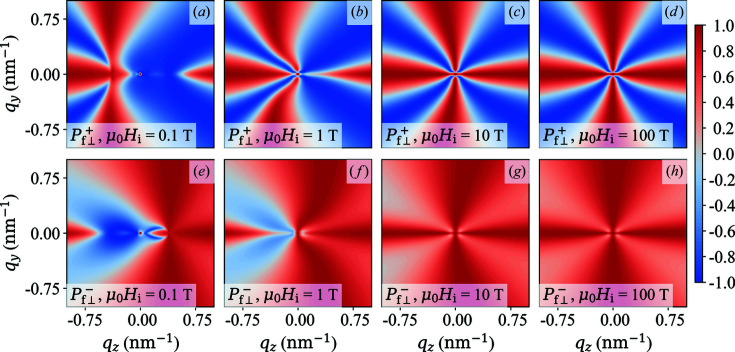
Effect of the DMI. Plot of 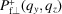 (upper row) and 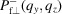 (lower row) as a function of 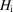 (see insets). 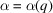 [equation (63)], 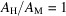 , 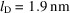 .

**Figure 15:**
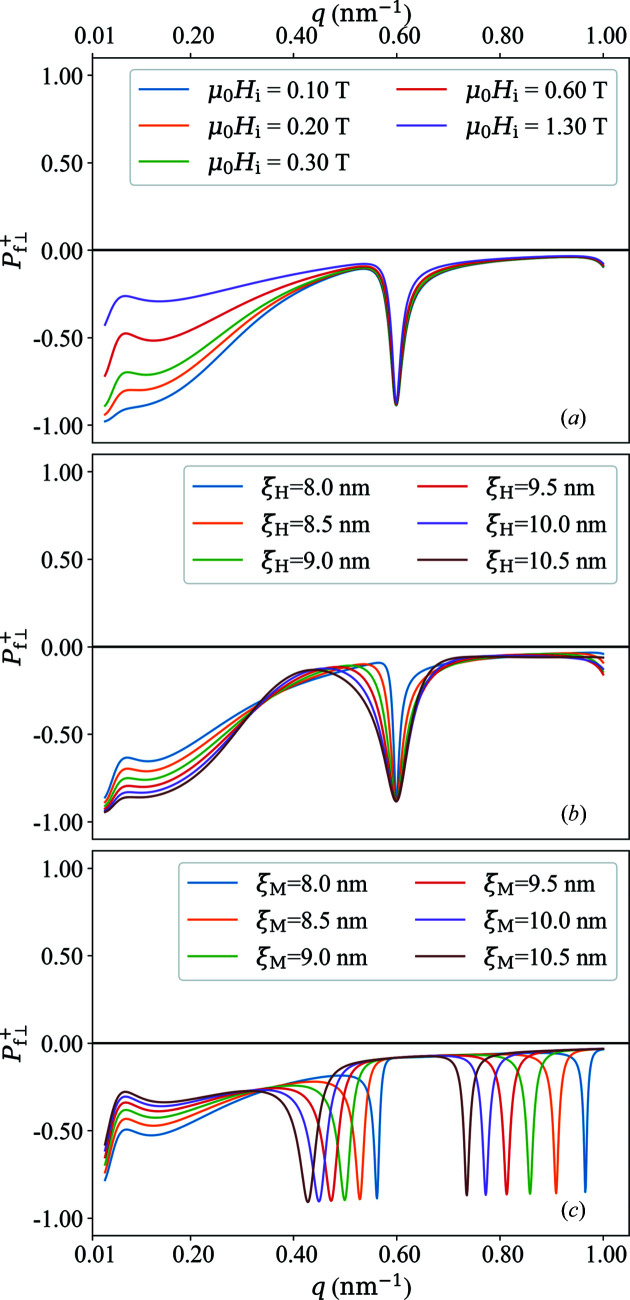
Results for the azimuthally averaged 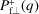 using the sphere form factor (instead of Lorentzian-squared functions) for both 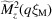 and 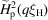 . (*a*) Field dependence (see inset) of 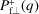 for 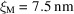 and 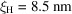 . (*b*) 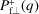 at 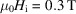 , 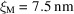 , but for increasing 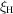 (see inset). (*c*) 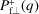 at 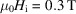 , 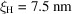 , but for increasing 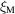 (see inset). 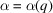 [equation (63)], 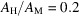 , 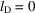 .

## Appendix A Non-spin-flip, spin-flip and SANSPOL cross sections

In this appendix, we present the expressions for the polarized SANS cross sections in terms of the Fourier components  of the magnetization. The two non-spin-flip and the two spin-flip POLARIS cross sections carry, respectively, the superscripts  and  and  and  , and the subscripts  and  refer to the respective scattering geometry (compare Fig. 1 ▸) (Michels, 2014 ▸):

where  , and the chiral function  is given by

Note that  for  . The two SANSPOL cross sections  =  +  and  =  +  read

The magnetic–magnetic and nuclear–magnetic cross terms have been abbreviated as follows:

In actual SANSPOL and POLARIS experiments the neutron optics (polarizer, spin flipper, analyzer) do not work perfectly and polarization corrections become necessary. The incident beam polarization efficiency may be denoted by  , where  are, respectively, the number of neutrons with spins aligned antiparallel and parallel with respect to  ; note that  for an unpolarized beam. The efficiency of the spin flipper is  with  for flipper off and  for flipper on. We emphasize that the half-polarized SANSPOL cross sections  and  can be obtained directly and corrected for nonideal neutron polarization provided that the parameters  and ε are known from reference measurements. For POLARIS, it is necessary to measure all four partial cross sections  ,  ,  and  in order to correct for spin leakage between the different channels (Wildes, 2006 ▸). Such corrections can *e.g.* be accomplished by means of the *BerSANS* (Keiderling, 2002 ▸; Keiderling *et al.*, 2008 ▸), *Pol-Corr* (Krycka *et al.*, 2012 ▸) and *GRASP* (Dewhurst, 2021 ▸) software tools.

Moreover, we note that  for many polycrystalline bulk ferromagnets (Honecker *et al.*, 2010 ▸). However, in our theoretical treatment we explicitly take into account the polarization dependence of the SANSPOL and spin-flip cross sections via the chiral function  . This is relevant *e.g.* for systems where inversion symmetry is broken and the DMI is operative (Michels *et al.*, 2019 ▸; Quan *et al.*, 2020 ▸).

## Appendix B Selected results for the polarization of the scattered neutrons of bulk ferromagnets

In this appendix we provide some selected graphical representations for the dependency of the polarization of the scattered neutrons on the magnitude and orientation of the scattering vector, the applied magnetic field, the ratio of  to  , the ratio of nuclear to longitudinal magnetic scattering, and the DMI (via the exchange length  ). Only results for  are shown. The following materials parameters are used: *A* = 4.7 pJ m^−1^;  1.64 T (  2.1 nm); *D* = 2 mJ m^−2^ (  1.9 nm) (Honecker *et al.*, 2013 ▸).

Fig. 10 ▸ shows the two-dimensional final polarization  and Fig. 11 ▸ depicts the corresponding  azimuthally averaged data  for different values of the applied magnetic field  ,  [equation (63)],  ,  . The local extrema in  at small *q* are due to  ; setting  constant results in smooth and continuously decaying functions. Figs. 12 ▸ and 13 ▸ display the polarization  for different ratios of  (Fig. 12 ▸) and for different (constant) α values (Fig. 13 ▸) at a constant field of  0.3 T and for  . Including the DMI results in asymmetric  patterns at nonsaturating fields (see Fig. 14 ▸). For the calculation of  according to equation (44*a* [Disp-formula fd44a]), we have up to now assumed Lorentzian-squared functions for  and  [compare equations (41)–(42)] with   . The effect of a hard-sphere form factor for  and  with different values for  and  is depicted in Fig. 15 ▸. Here, peak-type features may appear in  , which might be detected in highly monodisperse particulate systems.
